# Supplementary material for: Apical cell expansion maintained by Dusky-like establishes a scaffold for corneal lens morphogenesis
Source: Sci Adv. 2024 Aug 21;10(34):eado4167. doi: 10.1126/sciadv.ado4167 (PMC11338227; doi:10.1126/sciadv.ado4167)
Supplement: Supplementary file 1 — Figs. S1 to S6 Legend for data S1 [file sciadv.ado4167_sm.pdf]

Science Advances
AAAS

Supplementary Materials for

Apical cell expansion maintained by Dusky-like establishes a scaffold for corneal lens morphogenesis

Neha Ghosh and Jessica E. Treisman

Corresponding author: Jessica E. Treisman, jessica.treisman@nyulangone.org

Sci. Adv. **10**, eado4167 (2024)
DOI: 10.1126/sciadv.ado4167

The PDF file includes:

Figs. S1 to S6
Legend for data S1

Other Supplementary Material for this manuscript includes the following:

Data S1

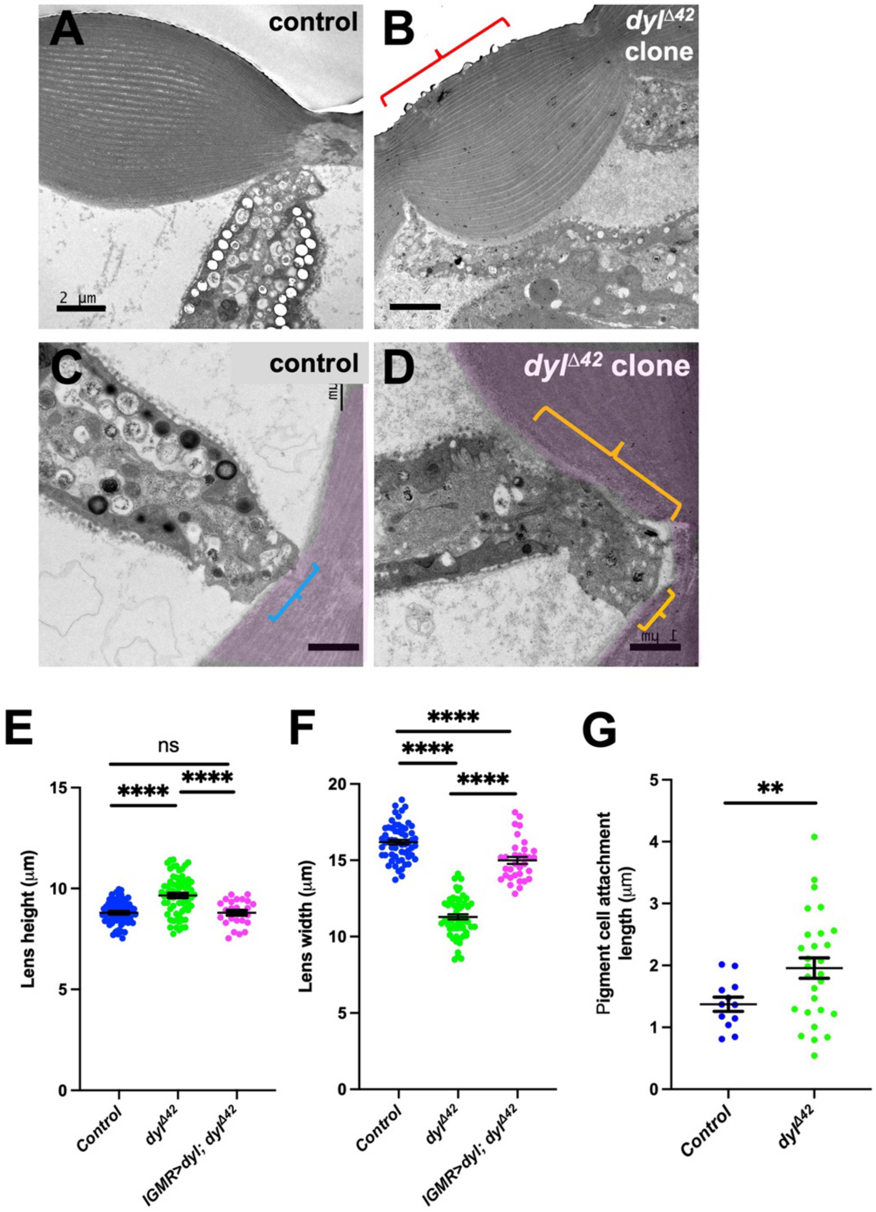

Fig. S1. Changes in corneal lens morphology in *dyl* mutants. (**A-D**) transmission electron micrographs of wild-type (**A, C**) and *dyl*$^{\Delta 42}$ (**B, D**) ommatidia in adult eyes. (**B**) shows defects and protrusions on the external surface of the *dyl* mutant corneal lens (bracket). (**C, D**) show sites of contact (brackets) between the pigment cells and the corneal lens (pseudocolored in magenta). Scale bars: 2 µm (**A, B**), 1 µm (**C, D**). (**E, F**) graphs showing corneal lens height (**E**) and corneal lens width (**F**) in wild type control regions of mosaic adult eye sections (n=73/11), *dyl*$^{\Delta 42}$ clones (n=68/11), and *dyl*$^{\Delta 42}$ clones expressing *UAS-dyl* with *lGMR-GAL4* (n=37/6). (**G**) Graph showing the length of pigment cell-corneal lens contacts in electron micrographs of control retinas (n=12/2) and *dyl*$^{\Delta 42}$ mutant clones (n=28/1). For all graphs, error bars show mean ± SEM. ****$p < 0.0001$, **$p=0.0061$, ns $p=0.993$, unpaired two-tailed t test with Welch's correction.

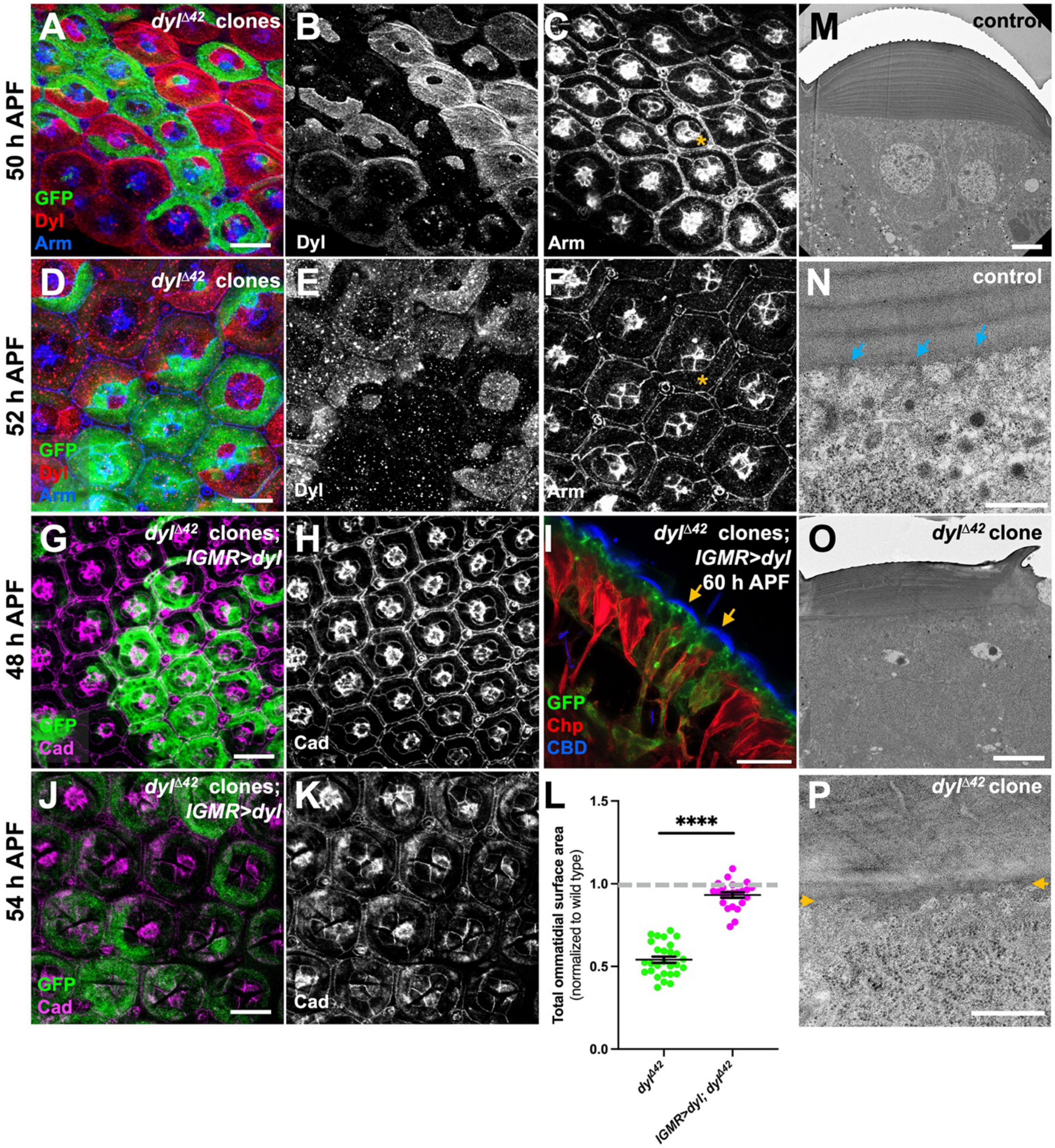

Fig. S2. Progression of the *dyl* mutant phenotype and rescue by UAS-*dyl*. (**A-F**) Retinas containing *dyl*$^{\Delta 42}$ clones marked with GFP (green), stained with anti-Dyl (**B, E**, red in **A, D**) and anti-Arm (**C, F**, blue in **A, D**). (**A-C**) 50 h APF; (**D-F**) 52 h APF. Dyl staining is absent from *dyl* mutant clones and is strongest at 50 h APF. Apical constriction of some *dyl* mutant central cells is marked with yellow asterisks. (**G, H, J, K**) Pupal retinas in which *dyl*$^{\Delta 42}$ clones expressing *UAS-dyl* with *lGMR-GAL4* are marked with GFP (green), stained with anti-E-cadherin (Ecad) and anti-N-cadherin (Ncad) to mark apical cell junctions (**H, K**, magenta in **G, J**). (**G, H**) 48 h APF; (**J, K**) 54 h APF. (**I**) Horizontal cryosection of a 60 h APF retina in which *dyl*$^{\Delta 42}$ clones

expressing *UAS-dyl* with *lGMR-GAL4* are marked with GFP (green, yellow arrows). The corneal lens is labeled with CBD (blue) and photoreceptors with anti-Chp (red). Expression of wild-type Dyl rescues the apical constriction and apical-basal contraction. (**L**) A graph depicting the total ommatidial apical surface area at 54 h APF in $dyl^{\Delta 42}$ clones (n=27/7) and $dyl^{\Delta 42}$ clones rescued with *lGMR-GAL4* driving *UAS-dyl* (n=22/8) normalized to internal control ommatidia (n=20/7). *UAS-dyl* fully rescues apical constriction in $dyl^{\Delta 42}$ mutant clones. Error bars show mean +/-SEM. ****$p < 0.0001$, unpaired two-tailed t test with Welch's correction. (**M-P**) transmission electron micrographs of 72 h APF corneal lenses from *dyl* mosaic eyes. (**M, N**) control; (**O, P**) *dyl* mutant. Darkly stained indentations in the apical plasma membrane of control ommatidia appear to connect to fibers in the cytoplasm (blue arrows), but clear sites of adhesion are not visible in *dyl* mutant ommatidia (yellow arrows indicate the junction between the corneal lens and the underlying cells). Scale bars: 10 µm (**A-H, J, K**), 20 µm (**I**), 2 µm (**M, O**), 500 nm (**N, P**).

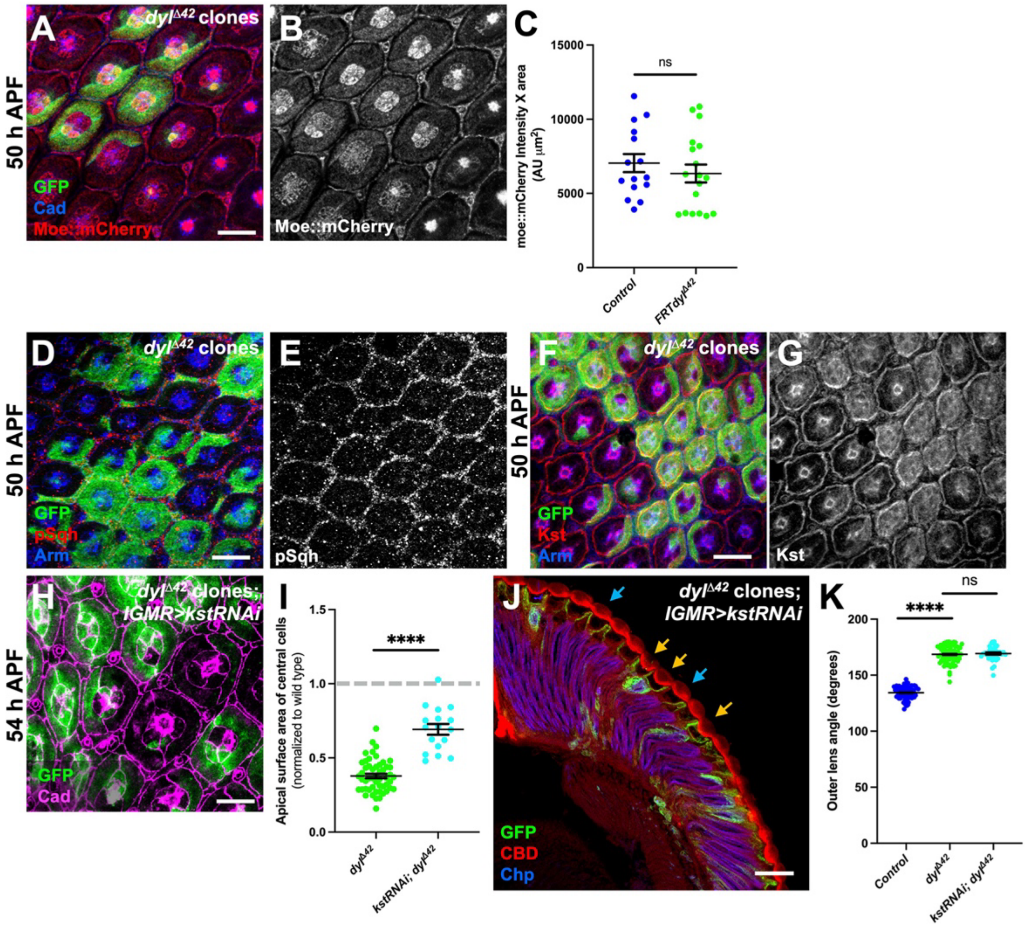

Fig. S3. Knocking down *kst* in *dyl* mutant clones does not rescue corneal lens curvature. (**A, B**) 50 h APF retinas with $dyl^{\Delta 42}$ clones marked with GFP (green), stained for Moe::mCherry (**B**, red in **A**) and Ecad and Ncad (blue in **A**). (**C**) Graph showing Moe::mCherry intensity per ommatidium multiplied by ommatidial area for *dyl* mutant (n=18/11) and control wild-type ommatidia. (n=15/11) ns p=0.4183, unpaired two-tailed t test with Welch's correction. The total amount of apical Moe:mCherry is not altered in *dyl* mutant cells. (**D-G**) 50 h APF retinas with $dyl^{\Delta 42}$ clones marked with GFP (green), stained for Arm (blue in **D, F**), pSqh (**E**, red in **D**) or anti-β_H-spectrin/Kst (**G**, red in **F**). *dyl* mutant clones accumulate β_H-spectrin at this stage, but do not show a change in pSqh. (**H**) 54 h APF retina with $dyl^{\Delta 42}$ mutant clones expressing *UAS-kst RNAi* with *lGMR-GAL4*, marked with GFP (green) and stained for Ecad and Ncad (magenta). (**I**) Graph showing the apical surface area of central cells in $dyl^{\Delta 42}$ mutant clones (data from **Fig. 2G**) and $dyl^{\Delta 42}$ mutant clones expressing *kst RNAi* (n=16/7) normalized to internal control ommatidia at 54 h APF. (**J**) Cryosection of an adult eye expressing *kst* RNAi in *dyl* mutant clones marked with GFP (green), stained with anti-Chp (blue) and CBD (red). *kst* knockdown fails to rescue the corneal lens shape (yellow arrows) compared to wild-type corneal lenses (blue arrows). Scale bars: 10 μm (**A, D, F, H**), 20 μm (**J**). (**K**) Graph showing the outer angle between adjacent corneal lenses in control, $dyl^{\Delta 42}$ mutant clones (data from **Fig. 1J**), and *kst RNAi* in $dyl^{\Delta 42}$ mutant clones (n=35/6). Although knocking down *kst* in $dyl^{\Delta 42}$ mutant clones significantly rescues the apical surface area of central cells, it is not sufficient to restore normal corneal lens curvature. Error bars show mean +/-SEM. ****$p < 0.0001$, ns $p = 0.4159$, one-sample t and Wilcoxon test (**I**) or unpaired two-tailed t test with Welch's correction (**K**).

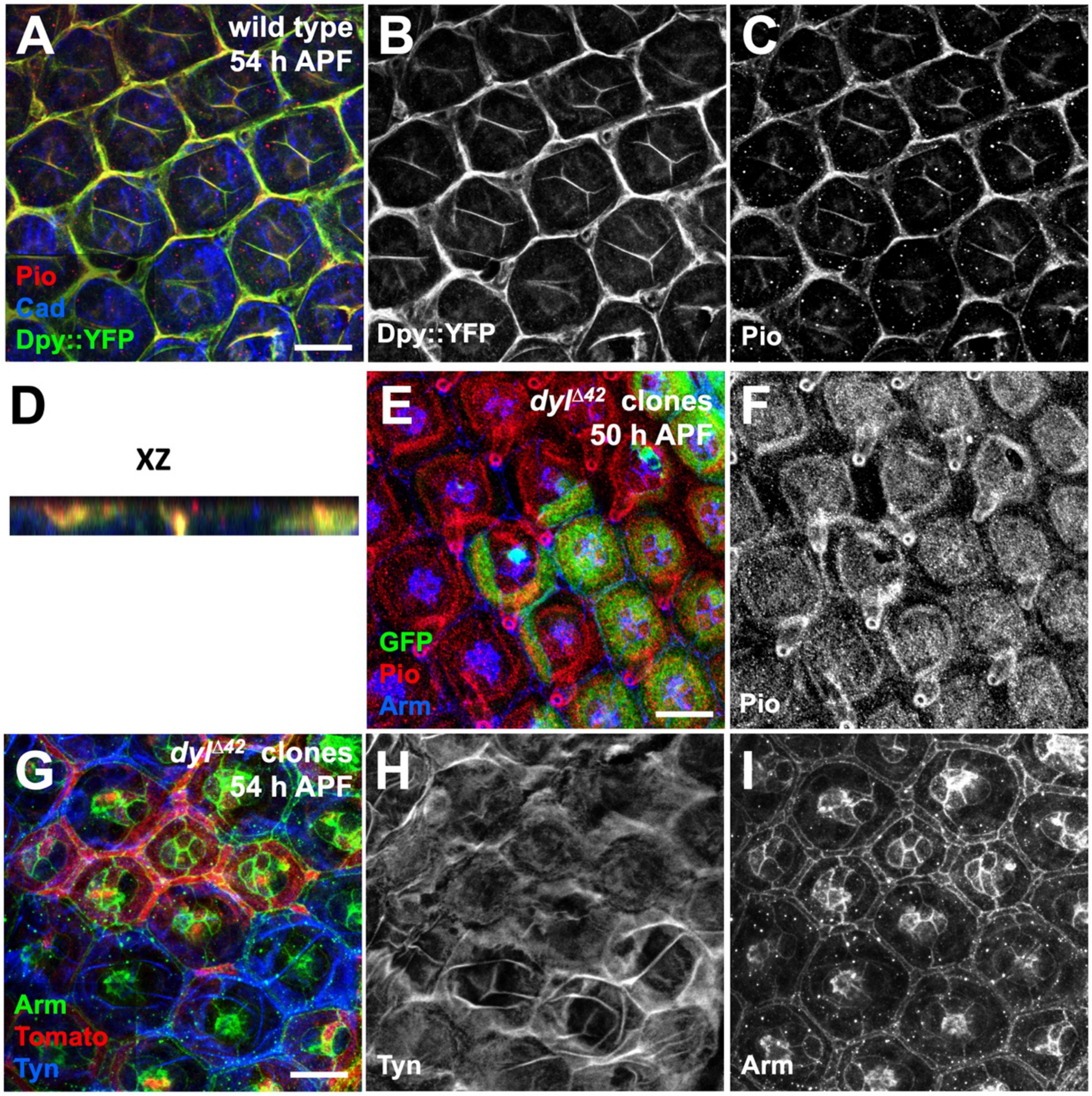

Fig. S4. Multiple ZP-domain proteins form an organized structure at the apical surface of the retina. (**A-D**) A 54 h APF pupal retina showing colocalization of Dpy-YFP (**B,** green in **A, D**) and Pio (**C**, red in **A, D**). Ecad and Ncad (blue) mark the apical junctions. An orthogonal section (**D**, apical up) shows that these proteins are in the same plane, apical to cadherin. (**E-I**) Pupal retinas containing *dyl*$^{\Delta 42}$ clones marked with GFP (green, **E**) or Tomato (red, **G**), stained with anti-Arm (**I**, blue in **E**, green in **G**), anti-Pio (**F**, red in **E**), or anti-Tyn (**H**, blue in **G**). (**E, F**) 50 h APF; (**G-I**) 54 h APF. Pio organization is not affected in *dyl*$^{\Delta 42}$ clones at 50 h APF; Tyn shows a filamentous organization in wild-type but not *dyl* mutant ommatidia at 54 h APF. Arm intensity is increased in *dyl* mutant clones. Scale bars: 10 μm.

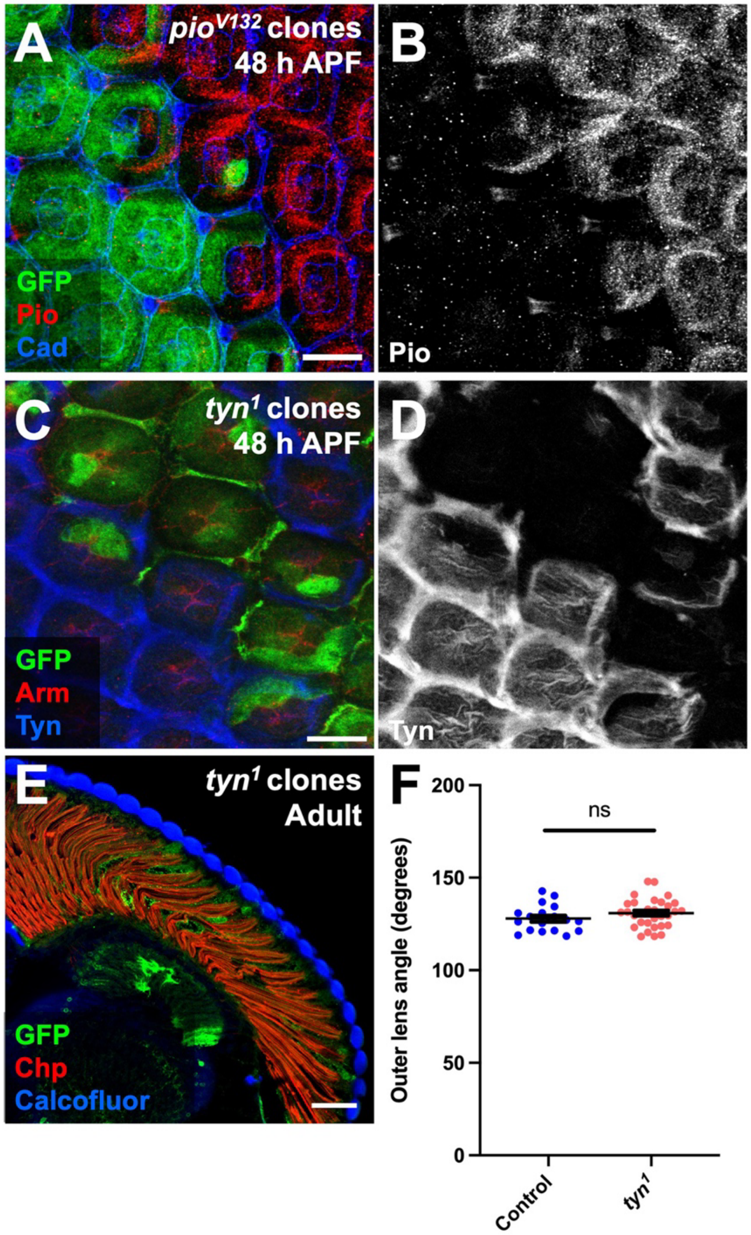

Fig. S5. Loss of *tyn* does not alter corneal lens shape. (**A-D**) *pio*^{V132} mutant clones (**A, B**) or *tyn*^{1} mutant clones (**C, D**) marked with GFP (green) in 48 h APF pupal retinas labeled with anti-Ecad and anti-Ncad (blue in **A)** or anti-Arm **(**red in **C**) and anti-Pio (**B**, red in **A**) or anti-Tyn (**D**, blue in **C**). Both alleles are protein nulls. (**E**) Horizontal cryosection of an adult eye with *tyn*^{1} mutant clones marked with GFP (green), labeled with anti-Chp (red) and Calcofluor White (blue). (**F**) A graph illustrating the outer lens angle between neighboring corneal lenses in internal wild type control (n=18/6) and *tyn*^{1} mutant clones (n=31/6) in the adult eye. Error bars show mean +/-SEM. ns p = 0.185. Scale bars: 10 µm (**A, C**), 20 µm (**E**).

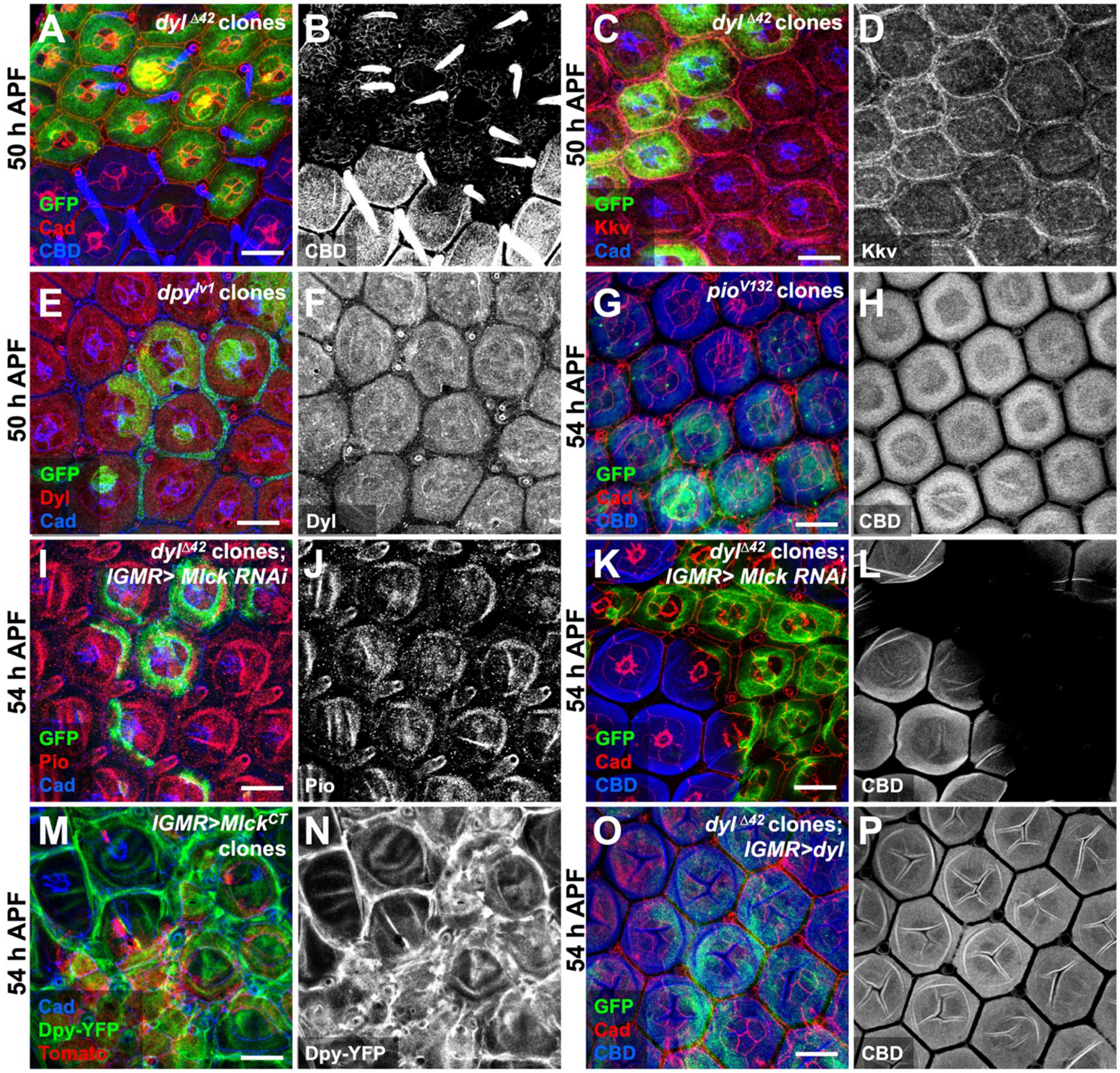

Fig. S6. Apical constriction acts on chitin through ZP-domain proteins. (**A-D**) 50 h APF pupal retinas with *dyl*$^{\Delta 42}$ clones marked with GFP (green), stained with CBD (**B,** blue in **A**), anti-Kkv (**D**, red in **C**), and anti-Ecad and Ncad (red in **A**, blue in **C**). At 50 h APF there is a small amount of chitin above the *dyl*$^{\Delta 42}$ clones compared to the more robust chitin accumulation above wild-type ommatidia, but Kkv is still present at the apical surface of *dyl* mutant clones. CBD also labels mechanosensory bristles. **(E, F)** 50 h APF pupal retina with *dpy*^{lv1} mutant clones marked with GFP (green), stained with anti-Dyl **(F**, red in **E)** and anti-Ecad and Ncad (blue). Dyl localization is not affected in *dpy* clones. **(G, H)** 54 h APF pupal retina with *pio*^{V132} mutant clones marked with GFP (green), stained with CBD (**H**, blue in **G**) and anti-Ecad and Ncad (red). Loss of *pio* does not affect chitin accumulation at this stage. (**I-L**) *dyl*$^{\Delta 42}$ mutant clones expressing *UAS-Mlck RNAi* with *lGMR-GAL4*, marked by GFP (green) and stained with anti-Pio (**J**, red in **I**), CBD (**L**, blue in **K**) and anti-Ecad and Ncad (blue in **I**, red in **K**). Reduction of apical constriction in *dyl*$^{\Delta 42}$ clones by knocking down *Mlck* does not rescue Pio organization or

loss of chitin. (**M, N**) 54 h APF retina with clones expressing *UAS-Mlck*^{CT} with *lGMR-GAL4* marked with Tomato (red), stained for Dpy-YFP (**N**, green in **M**) and anti-Ecad and anti-Ncad (blue). Apical constriction is sufficient to alter Dpy organization. (**O, P**) 54 h APF retina with *dyl*$^{\Delta 42}$ clones expressing *UAS-dyl* with *lGMR-GAL4* marked with GFP (green) stained with CBD (**P**, blue in **O**) and Ecad and Ncad (red). Wild type *dyl* rescues chitin accumulation in *dyl*$^{\Delta 42}$ mutant ommatidia. Scale bars: 10 µm.

Data S1. Source data for graphs. This file has the raw data for the graphs shown in Fig. 1J, K; Fig. 2G, H, K; Fig. 3M, O; Fig. 4E, F; Fig. 6I-K; Fig. S1E-G; Fig. S2L; Fig. S3C, I, K; and Fig. S5F.
